# Determinants of tobacco use transitions in smoker nursing students in Catalonia: A prospective longitudinal study

**DOI:** 10.18332/tid/189484

**Published:** 2024-07-08

**Authors:** Kenza Laroussy, Esteve Fernández, Yolanda Castellano, Marcela Fu, Antoni Baena, Ariadna Feliu, Armando Peruga, Mercè Margalef, Olena Tigova, Jordi Galimany, Montserrat Puig, Carmen Moreno, Albert Bueno, Antonio López, Judith Roca, Judith Saura, Cristina Martínez

**Affiliations:** 1 WHO Collaborating Centre for Tobacco Control, Institut Català d'Oncologia – ICO, Barcelona, Spain; 2 Tobacco Control Research Group, Institut d’Investigació Biomèdica de Bellvitge – IDIBELL, Barcelona, Spain; 3 School of Nursing, University of Barcelona, Barcelona, Spain; 4 School of Medicine and Health Sciences, University of Barcelona, Barcelona, Spain; 5 Centro de Investigación Biomédica en Red de Enfermedades Respiratorias (CIBERES), Madrid, Spain; 6 Department of e-Health, School of Health Sciences, Universitat Oberta de Catalunya, Barcelona, Spain; 7 Center for Epidemiology and Health Policy, Faculty of Medicine, Clínica Alemana, Universidad del Desarrollo, Santiago, Chile; 8 Nursing care management, Equip d'Atenció Primària de Roses, Institut Català de Salut, Girona, Spain; 9 Nursing care management, Equip d'Atenció Primària - Valls Urbà, Institut Català de Salut, Tarragona, Spain; 10 Department of Nursing and Physiotherapy, School of Nursing and Physiotherapy, University of Lleida, Lleida, Spain; 11 Philip R. Lee Institute for Health Policy Studies, University of California San Francisco, San Francisco, United States

**Keywords:** longitudinal study, nursing students, tobacco use, tobacco use cessation, transition

## Abstract

**INTRODUCTION:**

The use of emerging tobacco and nicotine products affects tobacco use behaviors among college students. Thus, we aimed to examine transitions in tobacco use patterns and identify their predictors among smokers in a cohort of nursing students in Catalonia (Spain).

**METHODS:**

We conducted a prospective longitudinal study of Catalan nursing students between 2015–2016 and 2018–2019. We examined transitions in tobacco use patterns between baseline and follow-up among smokers from: 1) daily to non-daily smoking, 2) non-daily to daily smoking, 3) cigarette-only use to poly-tobacco use, 4) poly-tobacco use to cigarette-only use, 5) between products, 6) reducing consumption by ≥5 cigarettes per day (CPD); and 7) quitting smoking. We applied a Generalized Linear Model with a log link (Poisson regression) and robust variance to identify predictors of reducing cigarette consumption by ≥5 CPD and quitting smoking, obtaining both crude and adjusted (APR) prevalence ratios and their 95% confidence intervals (CIs).

**RESULTS:**

Among daily smokers at baseline, 12.1% transitioned to non-daily smoking at follow-up, while 36.2% of non-daily smokers shifted to daily smoking. Among cigarette-only users, 14.2% transitioned to poly-tobacco use, while 48.4% of poly-tobacco users switched to exclusive cigarette use. Among all smokers (daily and non-daily smokers), 60.8% reduced their cigarette consumption by ≥5 CPD and 28.3% quit smoking. Being a non-daily smoker (APR=0.33; 95% CI 0.19–0.55) and having lower nicotine dependence (APR=0.78; 95% CI 0.64–0.96) were inversely associated with reducing cigarette consumption, while being a non-daily smoker (APR=1.19; 95% CI: 1.08–1.31) was directly associated with quitting smoking.

**CONCLUSIONS:**

Nursing students who smoked experienced diverse transitions in tobacco use patterns over time. Evidence-based tobacco use preventive and cessation interventions are needed to tackle tobacco use among future nurses.

## INTRODUCTION

Tobacco use continues to be a primary public health concern, being the cause of 8.7 million deaths globally every year^1^. Despite efforts to control tobacco use, the emergence of novel tobacco and nicotine products, new forms of targeted tobacco advertising, and renewed tobacco industry activity have led to changes in smoking behaviors among specific subgroups such as young adults, including college students^1-3^.

The American College Health Association (ACHA) estimated that by 2023, almost 40% of college students in the United States were regularly using tobacco and nicotine products in the past three months^2^. In particular, 24.2% were daily users, 8.9% were weekly users, and 7.2% were monthly users^2^. The most commonly used product was electronic cigarettes (e-cigarettes), whereas in Europe, the most common product used is cigarettes^4^. During the last years, there has been a marked increase in the prevalence of experimentation with tobacco and nicotine products, alternative tobacco product use, and poly-tobacco use among college students^2,5,6^. Current data reveals that nearly 59% of college students have ever used at least one tobacco or nicotine product, with 41% having ever used two or more tobacco or nicotine products^5^*.* Furthermore, while the prevalence of regular use of alternative or novel tobacco products varies significantly by country, it is becoming increasingly popular among both college smokers and non-smokers^6^. In addition, the use of multiple tobacco products among this group and their combination with other substances, such as cannabis, is worrying, with prevalence rates of concern (up to 9%, according to Odani et al.^7^).

Both experimenting and regularly using alternative tobacco products may lead to cigarette initiation among non-smokers, increasing the likelihood of transitioning to regular cigarette users^5,8-10^. Moreover, they increase the probability of poly-tobacco use among cigarette users, which can lead to higher levels of nicotine intake and greater nicotine addiction and hamper quitting smoking^8,9,11^.

The university period is a crucial time for most college students to establish smoking behaviors^3^, emphasizing the need to identify predictors and correlates of changes in tobacco use. This issue is particularly important among nursing students, who should perform tobacco prevention and cessation interventions in their future professional roles. Despite their committed role in tobacco control, in Spain, nursing students have a high prevalence of tobacco use (35.1%)^12^, which in most cases is similar to or even higher than that reported in the general population^13^. It is also of concern that the prevalence of tobacco use among nursing students is generally higher than that of other health science students, such as medical students, who have a prevalence of smoking of 17.5%^12^. Therefore, this study aimed to examine changes in tobacco use patterns and identify their predictors among smokers of a cohort of nursing students.

## METHODS

### Design and participants

We conducted a prospective longitudinal study of a sample of nursing students from all nursing schools in Catalonia, Spain, from the academic year 2015–2016 to the academic year 2018–2019. At baseline, 4381 nursing students completed a questionnaire after signing an informed consent form. They agreed to participate in the study, including their willingness to participate in future follow-ups. The description, participation, and data collection of baseline and follow-up studies have already been reported^14-16^. For this study, the inclusion criteria were completing the baseline and follow-up surveys and being current smokers (daily or non-daily) at baseline.

### Instrument and variables

At baseline, participants completed a self-administered paper-and-pencil questionnaire that assessed the use of different tobacco products, e-cigarettes, heated tobacco products (HTPs), and cannabis. The Global Health Professional Survey (GHPS) was used to create the questionnaire. For follow-up, the baseline questionnaire was a model for launching an online version through the LimeSurvey platform. Before the survey launch, we conducted a pilot test of the follow-up questionnaire with 20 collaborating researchers and 50 study participants (further details available elsewhere)^15^.

In both the baseline and follow-up questionnaires, we asked participants about their current and past use of various tobacco products, including manufactured (MF) and roll-your-own (RYO) cigarettes, cigars/cigarillos/little cigars and waterpipes, e-cigarettes, HTPs, and cannabis. We used the Centers for Disease Control and Prevention and ‘*Diagnostic and Statistical Manual of Mental Disorders Fourth Edition*’ definitions of smoking behavior to classify participants according to their tobacco use^17^. Participants who were using combustible tobacco products at the time of the survey (either MF or RYO cigarettes) were considered current smokers. Participants who were using any of these products daily were classified as daily smokers, while those who were using them not every day but at least once in the last 30 days were classified as non-daily smokers.

Current smokers were asked about their tobacco use patterns, including age at smoking initiation (<17 and ≥17 years); reasons why they started smoking (because my friends/classmates smoked, because one of my family members smoked, because my teachers smoked, to experiment with new experiences, because it is trendy, to feel older, to meet people or to flirt, and other); reasons why they currently smoke (for weight control, to reduce stress/relax, for socializing, because my friend/family smokes, because it is trendy, for pleasure, because I could not quit, and other); number of cigarettes per day (classified as <10, 10–19, and ≥20); time (in minutes) to first cigarette after waking up (≤5, 6–30, 31–60, or >60); if they have seriously tried to quit smoking in the last year (yes or no); number of attempts to quit of at least 24 hours in the last year (1 or ≥2); and if they have the intention to quit or cut back in the following year (yes or no).

We used cigarettes per day (CPD) smoked and time to first cigarette (TFC) to calculate the heaviness of the smoking index (HSI) to describe their nicotine dependence. The score was categorized as: <10 CPD, 1 point (p); 10–19 CPD, 2 p; CPD ≥20, 3 p; and TFC: ≥5, 3 p; 6–30, 2 p; 31–60, 1 p; or >60, 0 p. By summing the scores from both variables, we obtained a score between 0 and 6 and considered an HSI from 0–2 as ‘low nicotine dependence’, 3–4 as ‘medium nicotine dependence’, and 5–6 as ‘high nicotine dependence’^18^.

In addition, at baseline and follow-up, sociodemographic characteristics of all participants were collected. Baseline sociodemographic characteristics included sex (male, female); age (≤19, 20–24, or ≥25 years); year of degree (first, second, third, or fourth year); place of birth (Catalonia or outside of Catalonia); location of the nursing school (Barcelona or outside of Barcelona), and type of university (public, private with public funding, or private). At follow-up, we ascertained whether they had finished the nursing degree (yes or no); occupation (nursing student, nurse, or other); year of degree for continuing students (second, third, or fourth); work area for recently graduated employed nurses (hospital or other) and type of institution they worked (public, private, and private with public funding); if they were living with family or were independent; household monthly income (€) (≤1500, 1501–3000, or >3000); and marital status (single, married or cohabiting, divorced, or widowed).

The main dependent variable was tobacco use transition between baseline and follow-up. Seven transitions were established: 1) from daily to non-daily smoking; 2) from non-daily to daily smoking; 3) from cigarette-only use (only MF and/or RYO cigarettes) to poly-tobacco use (MF and/or RYO cigarettes with other product/s); 4) from poly-tobacco use to cigarette-only use; 5) between products; 6) reduce cigarette consumption by ≥5 CPD; and 7) quit smoking. Participants who did not change their tobacco use patterns between the two surveys were defined as: continued as a daily smoker, continued as a non-daily smoker, continued as a cigarette-only user, continued as a poly-tobacco user, or continued as a current smoker. Those who reduced cigarette consumption by ≥5 CPD were compared with those who reduced their consumption by <5 CPD, increased the number of consumed cigarettes, or did not change the number of CPD.

The independent variables included those related to the tobacco use pattern and sociodemographic characteristics at baseline.

### Ethical considerations

The study protocol was approved by the Ethics Committee of the Hospital Universitari de Bellvitge (PR239/18). Informed consent was obtained from all individual participants at baseline and follow-up.

### Statistical analysis

For the descriptive analysis, we calculated the prevalence (%) and its 95% confidence intervals (CIs), and for the bivariate analysis, we used the chi-squared test. To analyze the predictors of reducing cigarette consumption and quitting smoking, we performed a multivariable Generalized Linear Model with a log link (Poisson regression) and robust variance to obtain both crude (PR) and adjusted prevalence ratios (APRs) and their 95% CI. For both transitions, the adjusted models included sex, baseline age, and the significant variables identified in the bivariate analysis, except the number of CPD since it was collinear with the baseline smoking status. Predictors of transition from daily to non-daily use, from non-daily to daily smoking, from cigarette-only use to poly-tobacco use, from poly-tobacco use to cigarette-only use, and between products, were not assessed due to the small number of participants that experienced these transitions. In addition, we conducted sex- and age-specific analyses to examine potential interactions by: 1) stratifying participants’ sociodemographic characteristics and tobacco use patterns variables by age and sex; 2) calculating the cumulative rates of transition from daily to non-daily smoking, from non-daily to daily smoking, from cigarette-only use to poly-tobacco use, from poly-tobacco use to cigarette-only use, cigarette consumption reduction ≥5 CPD, and quitting smoking stratified by sex and age; and 3) adding an interaction term with sex and age with the main independent variables in the regression models. All tests were two-tailed, and the statistical significance was p<0.05. All analyses were performed using the statistical package IBM SPSS Statistics version 25.

## RESULTS

### Description of the sample

At baseline, 4381 nursing students completed the survey. Of these, 1288 (29.7% of the sample) reported being current smokers, of whom 61.9% were daily smokers and 38.1% were non-daily smokers. Of all current smokers at baseline, 276 (21.4%) filled in the follow-up survey, with 198 (71.7%) continuing as current smokers while 78 (28.3%) had quit smoking. The percentages of daily and non-daily smokers at follow-up were 70.7% and 29.3%, respectively. Table 1 shows the baseline and follow-up characteristics of the cohort by sex and baseline smoking status. Of the participants followed, 241 (87.3%) were women, and 225 (82.4%) were aged ≤24 years at baseline. At follow-up, 103 (37.3%) were nursing students and 161 (58.3%) were nurses. There were no significant differences by sex among the participants. At baseline, participants aged ≤19 years were more likely to be non-daily smokers (47.1%), while those aged ≥20 years were more likely to be daily smokers (76.9%, p<0.001).

**Table 1:** Sociodemographic characteristics of the followed Catalan smoker nursing students at baseline (2015-2016) and follow-up (2018-2019) according to sex and baseline smoking status (N=276)

*Characteristics*	*Total*		*Sex*					*Baseline smoking status*				
			*Male*		*Female*			*Daily smoker*		*Non-daily smoker*		
	*n*	*%*	*n*	*%*	*n*	*%*	*p ^a^*	*n*	*%*	*n*	*%*	*p ^b^*
**Overall**	276	100	35	12.7	241	87.3		170	61.6	106	38.4	
**At baseline**												
**Age** (years)							0.044					**<0.001**
≤19	88	32.2	7	20.0	81	34.0		39	23.1	49	47.1	
20–24	137	50.2	17	48.6	120	50.5		94	55.6	43	41.4	
≥25	48	17.6	11	31.4	37	15.5		36	21.3	12	11.5	
**Year in nursing school**							0.482					0.125
First	99	36.4	10	29.4	89	37.4		56	33.1	43	41.7	
Second	67	24.6	8	23.5	59	24.8		38	22.5	29	28.2	
Third	59	21.7	7	20.6	52	21.8		43	25.5	16	15.5	
Fourth	47	17.3	9	26.5	38	16.0		32	18.9	15	14.6	
**Place of birth**							0.593					0.070
Catalonia	232	86.2	28	82.4	204	86.8		149	89.2	83	81.4	
Outside of Catalonia	37	13.8	6	17.6	31	13.2		18	10.8	19	18.6	
**Location of nursing school**							0.582					0.103
Barcelona	236	85.5	31	88.6	205	85.1		150	88.2	86	81.1	
Outside of Barcelona	40	14.5	4	11.4	36	14.9		20	11.8	20	18.9	
**Type of nursing school**							0.587					0.104
Public	91	33.0	9	25.7	82	34.0		48	28.2	43	40.6	
Private with public funding	66	23.9	10	28.6	56	23.2		43	25.3	23	21.7	
Private	119	43.1	16	45.7	103	42.8		79	46.5	40	37.7	
**At follow-up**												
**Finished nursing degree**							0.878					0.225
Yes	161	58.3	20	57.1	141	58.5		104	61.2	57	53.8	
No	115	41.7	15	42.9	100	41.5		66	38.8	49	46.2	
**Occupation**							0.782					0.177
Nursing students	103	37.3	14	40.0	89	36.9		58	34.1	45	42.5	
Nurses	161	58.3	20	57.1	141	58.5		104	61.2	57	53.8	
Other	12	4.4	1	2.9	11	4.6		8	4.7	4	3.7	
**Year in nursing school** (students)							0.729					0.931
Second or third	21	20.4	2	14.3	19	21.3		12	20.7	9	20.0	
Fourth	82	79.6	12	85.7	70	78.7		46	79.3	36	80.0	
**Work area** (nurses)							0.741					0.264
Hospital	115	82.1	14	77.8	101	82.8		74	79.6	41	87.2	
Other	25	17.9	4	22.2	21	17.2		19	20.4	6	12.8	
**Type of institution they work in** (nurses)							0.312					0.477
Public	66	47.1	6	33.3	60	49.2		46	49.5	20	42.6	
Other	74	52.9	12	66.7	62	50.8		47	50.5	27	57.4	
**Living status**							0.295					0.176
With family	157	61.1	18	52.9	139	62.3		92	57.9	65	66.3	
Independent	100	38.9	16	47.1	84	37.7		67	42.1	33	33.7	
**Household monthly income**												
(€)							0.722					0.213
≤1500	82	29.8	12	34.3	70	29.1		51	30.0	31	29.3	
1501–3000	81	29.3	11	31.4	70	29.0		53	31.2	28	26.4	
>3000	50	18.1	4	11.4	46	19.1		34	20.0	16	15.1	
Do not know/Did not answer	63	22.8	8	22.9	55	22.8		32	18.8	31	29.2	
**Marital status**							0.585					0.333
Single	181	70.7	22	66.7	159	71.3		109	68.6	72	74.2	
Other	75	29.3	11	33.3	64	28.7		50	31.4	25	25.8	

a Chi-squared test (male vs female).

b Chi-squared test (daily smoker vs non-daily smoker).

### Tobacco use patterns at follow-up

The majority of current smokers at follow-up, whether daily or non-daily, exclusively used MF and/or RYO cigarettes (76.2% and 67.9%, respectively), consumed <10 CPD (87.5% and 100%, respectively), and had low nicotine dependence (76.5% and 100%, respectively). Poly-tobacco use was more frequent among daily and non-daily smokers using cannabis (14.0% and 13.2%, respectively) and waterpipes (9.8% and 24.5%, respectively), with the latter being more frequent among non-daily smokers than among daily smokers (p=0.008). Non-daily smokers consumed, on average, fewer CPD than daily smokers (100% vs 55.6%, p<0.01). A higher proportion of daily smokers than non-daily ones reported intending to cut back their cigarette consumption (75.0% vs 47.6%, p<0.01) (Supplementary file Table S1). Additionally, participants who had completed their nursing degree (either those who were nurses or had other situations) had a greater proportion of cigarette-only use (66.2%); in contrast, those who were still nursing students had a higher prevalence of poly-tobacco use (68.6%) (p<0.001) (Supplementary file Table S2). Sex and age were not associated with any of the variables related to the tobacco use patterns.

### Tobacco use transitions between baseline and follow-up

**Figure 1:**
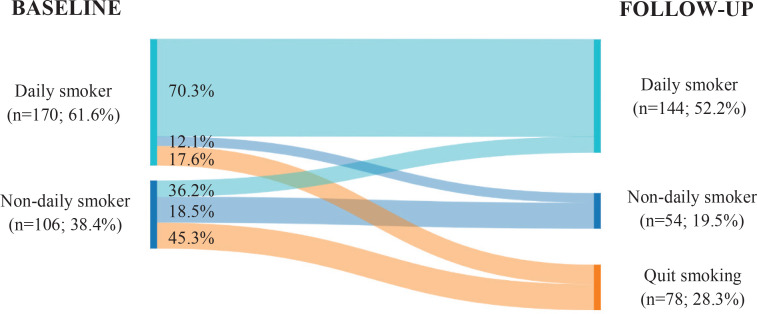
Transitions in tobacco use patterns among smokers of a cohort of Catalan nursing students from baseline (2015-2016) to follow-up (2018-2019) (N=276)

As presented in Table 2 and Figure 1, of all daily smokers at baseline, 12.1% transitioned to being non-daily smokers at follow-up. A high proportion of daily smokers with low nicotine dependence transitioned to non-daily smoking (19.5% vs 4.1%, p=0.012). Although there were no differences by type of product used, a product-by-product analysis showed a higher proportion of daily smokers who transitioned to being non-daily smokers among those who used cigarettes and cannabis concurrently (25% vs 10.8%, 2.5%, 6.7%, and 11.1%, p=0.011). Moreover, the lower the number of CPD, the greater the proportion of daily smokers who transitioned to being non-daily smokers (25.0%, 16.1%, and 3.4%, p<0.05). From the total of non-daily smokers at baseline, 36.2% (n=21) transitioned to being daily smokers at follow-up. Participants who had no intention to quit at baseline had a high proportion of non-daily smokers who transitioned to being daily smokers (46.5% vs 9.1%, p<0.05).

**Table 2:** Baseline sociodemographic characteristics and tobacco use patterns of Catalan nursing students who transitioned from non-daily to daily smoking and from daily to non-daily smoking from baseline (2015-2016) to follow-up (2018-2019) (N=276)

*Characteristics*	*Transitioned to daily smoking ^a^*			*Transitioned to non-daily smoking ^b^*		
	*n*	*%*	*p*	*n*	*%*	*p*
**Overall**	21	36.2		17	12.1	
**Sex**			0.160			0.523
Male	6	54.5		2	18.2	
Female	15	31.9		15	11.6	
**Age** (years)			0.681			0.198
≤19	11	42.3		6	18.8	
20–24	8	30.8		10	12.7	
≥25	2	40.0		1	3.6	
**Year in nursing school**			0.070			0.149
First	10	40.0		6	13.0	
Second	8	53.3		7	22.6	
Third	0	0		3	8.3	
Fourth	2	25.0		1	3.8	
**Age at smoking initiation**			0.702			0.513
<17	13	38.2		11	11.0	
≥17	8	33.3		6	15.0	
**Reasons why they started smoking^c^**						
Because my peer/family smoked	11	36.7	1.000	12	11.8	1.000
Other	16	36.4	1.000	9	9.4	0.166
**Reasons why they currently smoke^c^**						
To reduce stress/relax	11	44.0	0.408	10	10.9	0.586
For pleasure	14	40.0	0.579	14	13.2	0.571
Other	5	20.8	0.054	8	10.5	0.607
**Type of product used**			0.783			0.088
Cigarette-only use^d^	12	34.3		9	9.0	
Poly-tobacco use	9	39.1		8	20.0	
**Cigarettes per day**			0.105			**0.017**
<10	15	32.6		5	25.0	
10–19	6	60.0		10	16.1	
≥20	-			2	3.4	
**Heaviness of smoking index**			0.915			**0.012**
Low (0–2)	20	36.4		15	19.5	
Medium and high (3–6)	1	33.3		2	4.1	
**Quit attempts in the last year**			0.264			0.348
Yes	6	50.0		3	7.9	
No	15	32.6		14	13.7	
**Number of quit attempts**			0.294			0.718
1	3	75.0		1	6.3	
≥2	2	40.0		2	9.5	
**Are you seriously thinking about quitting now?**			**0.023**			0.055
Yes	1	9.1		4	26.7	
No	20	46.5		12	9.8	
**Are you thinking about cutting back consumption?**			0.783			0.859
Yes	10	35.7		10	11.2	
No	11	39.3		6	12.2	

a Compared with ‘continued as non-daily smokers’ (n=37).

b Compared with ‘continued as daily smokers’ (n=123).

c Multiple responses were accepted.

d Manufactured and/or roll-your-own cigarettes.

**Figure 2:**
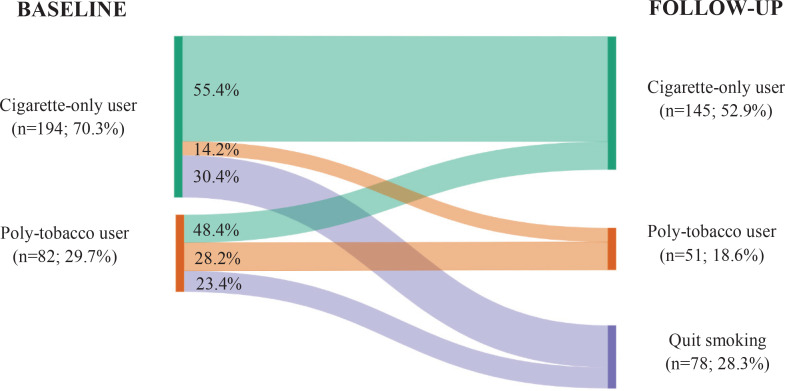
Transitions in type of tobacco use among smokers of a cohort of Catalan nursing students from baseline (2015-2016) to follow-up (2018-2019) (N=276)

Table 3 and Figure 2 present the transitions in the type of tobacco use between baseline and follow-up. Of all cigarette-only users at baseline, 14.2% had transitioned to poly-tobacco use at follow-up. In comparison to other age groups, a higher proportion (20.0%) of cigarette-only users aged 20–24 years at baseline shifted to poly-tobacco use at follow-up (p=0.027). Overall, 48.4% of poly-tobacco users at baseline transitioned to cigarette-only use. A higher proportion of poly-tobacco users switched to cigarette-only use among participants in the second and third years of their degree studies compared to those in the first and fourth years (76.9% and 75.0% vs 31.4% and 50.0%, p=0.012). Furthermore, compared with those who continued as poly-tobacco users, a lower proportion of participants who reported initiating smoking for reasons other than having a peer/family smoker transitioned to cigarette-only use (p=0.013).

**Table 3:** Baseline sociodemographic characteristics and tobacco use patterns of participants who transitioned from cigarette-only use to poly-tobacco use and from poly-tobacco use to cigarette-only use from baseline (2015-2016) to follow-up (2018-2019), in Catalan nursing students (N=276)

	*Transitioned to poly-tobacco use ^a^*			*Transitioned to cigarette-only use ^b^*		
	*n*	*%*	*p*	*n*	*%*	*p*
**Overall**	19	14.2		30	48.4	
**Sex**			0.053			0.418
Male	5	31.3		4	66.7	
Female	14	11.9		26	46.4	
**Age** (years)			**0.027**			0.212
≤19	5	15.2		9	36.0	
20–24	14	20.0		18	54.5	
≥25	0	0		3	75.0	
**Year in nursing school**			0.404			**0.012**
First	8	22.9		11	31.4	
Second	3	9.1		10	76.9	
Third	4	11.4		6	75.0	
Fourth	4	14.3		3	50.0	
**Age at smoking initiation** (years)			0.469			0.659
<17	14	15.7		21	46.7	
≥17	5	11.1		9	52.9	
**Reasons why they started smoking^c^**						
Because my peer/family smoked	16	17.4	0.115	22	55.0	0.160
Other	10	10.9	0.104	18	39.1	**0.013**
**Reasons why they currently smoke^c^**						
To reduce stress/relax	10	12.5	0.470	21	56.8	0.109
For pleasure	14	15.2	0.646	24	51.1	0.455
Other	12	17.4	0.272	12	38.7	0.127
**Smoking status**			0.399			0.160
Non-daily smoker	16	16.2		22	55.0	
Daily smoker	3	8.6		8	36.4	
**Cigarettes per day**			0.753			0.398
<10	6	13.6		12	57.1	
10–19	6	12.0		8	38.1	
≥20	7	17.5		10	55.6	
**Heaviness of smoking index**			0.920			0.871
Low (0–2)	12	14.0		21	47.7	
Medium and high (3–6)	7	14.6		9	50.0	
**Quit attempts in the last year**			0.423			0.176
Yes	7	17.9		7	70.0	
No	12	12.6		23	44.2	
**Number of quit attempts**			0.791			0.495
1	3	16.7		2	100	
≥2	4	20.0		4	80.0	
**Are you seriously thinking about quitting now?**			0.332			0.492
Yes	13	15.7		17	53.1	
No	6	12.5		12	41.4	
**Are you thinking about cutting back consumption?**			0.620			0.359
Yes	13	13.3		17	16.7	
No	6	9.2		12	16.9	

a Compared with ‘continued as cigarette-only users’ (n=115).

b Compared with ‘continued as poly-tobacco users’ (n=32).

c Multiple responses were accepted. Cigarette-only use includes manufactured and/or roll-your-own cigarettes.

**Figure 3:**
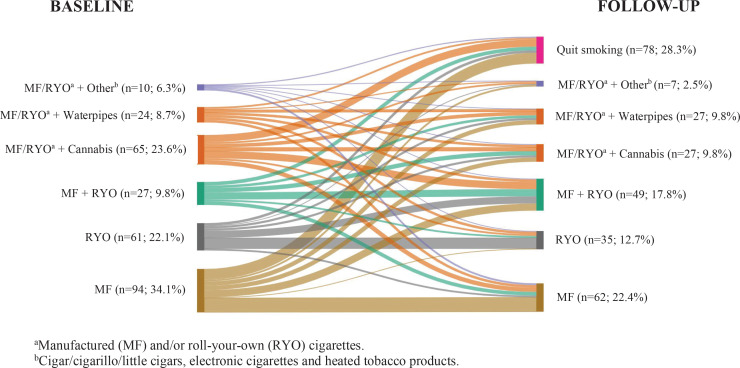
Transitions in tobacco product use among smokers of a cohort of Catalan nursing students from baseline (2015-2016) to follow-up (2018-2019) (N=276)

Figure 3 displays the product use between baseline and follow-up. At baseline, MF cigarettes (34.1%), MF and/or RYO cigarettes and cannabis (23.6%), and RYO cigarettes (22.1%) were the most commonly used products. At follow-up, the exclusive use of MF cigarettes continued to be the most common product used (22.4%); however, the prevalence of concurrent use of MF and/or RYO cigarettes with cannabis decreased to 9.8%, while MF and RYO cigarette use increased (17.8%). The exclusive use of RYO cigarettes continued as the third most common product used (12.7%). The prevalence of concurrent use of MF and/or RYO cigarettes and waterpipes slightly increased from 8.7% to 9.8% (13.6% considering only those who were current smokers at follow-up). Most poly-tobacco users of MF and/or RYO cigarettes and e-cigarettes or cigars/cigarillos/little cigars (70%) switched to using only MF cigarettes; however, the prevalence of HTPs use increased at follow-up. Finally, users of MF cigarettes at baseline had the highest percentage of quitters at follow-up (26%), whereas users of waterpipes had the lowest percentage of quitters (4.2%).

As shown in Table 4, 60.8% of current smokers (both daily and non-daily) at baseline reduced their cigarette consumption by ≥5 CPD at follow-up. The proportion of smokers who reduced their cigarette consumption was higher among participants aged ≥20 years compared with those aged ≤19 years (63.8% and 68.8% vs 43.9%, p=0.022); among participants who started smoking before the age of 17 years compared with those who started after the age of 17 years (64.7% vs 46.0%, p=0.013); among those who reported initiating smoking because they had a peer/family smoker compared with those who had not reported this reason (64.1% vs 35.9%, p=0.028); among those who reported continuing smoking to reduce stress or relax compared with those who not reported it (65.5% vs 34.5%, p=0.024); among those who were daily smokers compared with non-daily smokers (74.1% vs 21.1%, p<0.001); among those who smoked more CPD (89.5%, 77.8% and 12.3%, p<0.001); and among those with medium and high nicotine dependence compared with those with low dependence (83.1% vs 46.6%, p<0.01).

**Table 4:** Predictors of reducing cigarette consumption by ≥5 cigarettes/day in a cohort of Catalan nursing students from baseline (2015-2016) to follow-up (2018-2019) according to baseline sociodemographic characteristics and tobacco use patterns (N=276)

	*Reduced cigarette consumption by ≥5 cigarettes/day ^a^*				
	*n*	*%*	*p*	*APR ^b^*	*95% CI*
**Overall**	115	60.8			
**Sex**			0.676		
Male	12	54.5		1.26	0.92–1.73
Female	103	59.3		1.00	
**Age** (years)			**0.022**	1.00^c^	0.97–1.02
≤19	25	43.9			
20–24	67	63.8			
≥25	22	68.8			
**Year in nursing school**			0.805		
First	40	56.3			
Second	26	57.8			
Third	28	65.1			
Fourth	21	61.8			
**Age at smoking initiation** (years)			**0.013**		
<17	86	64.7		1.23	0.95–1.60
≥17	29	46.0		1.00	
**Reasons why they started smoking^d^**					
Having peer/family smoker	84	64.1	**0.028**	1.09	0.85–1.41
Other	82	59.0	0.887		
**Reasons why they currently smoke^d^**					
To reduce stress/relax	76	65.5	**0.024**	1.11	0.85–1.34
For pleasure	88	62.9	0.079		
Other	64	64.6	0.086		
**Smoking status**			**<0.001**		
Non-daily smoker	12	21.1		**0.33**	**0.19–0.55**
Daily smoker	103	74.1		1.00	
**Type of tobacco use**			0.542		
Cigarette-only use^e^	80	60.2			
Poly-tobacco use	35	55.6			
**Cigarettes per day**			**<0.001**		
<10	8	12.3			
10–19	56	77.8			
≥20	51	89.5			
**Heaviness of smoking index**			<0.001		
Low (0–2)	61	46.6		**0.78**	**0.64–0.96**
Medium and high (3–6)	54	83.1		1.00	
**Quit attempts in the last year**			0.802		
Yes	28	57.1			
No	87	59.2			
**Number of quit attempts**			0.805		
1		57.9			
≥2	16	61.5			
**Are you seriously thinking about quitting now?**			0.247		
Yes	13	50.0			
No	101	62.0			
**Are you thinking about cutting back consumption?**			0.523		
Yes	71	61.2			
No	43	56.6			

a Compared with those who reduced their consumption by <5 cigarettes/day, increased their consumption, or who did not change their consumption (n=81). APR: adjusted prevalence ratio.

b PR adjusted for sex, age group, age at smoking initiation, having started smoking because they have a family/peer smoker, current smoking to reduce stress/ relax, smoking status, and heaviness of smoking index.

c Continuous variable.

d Multiple responses were accepted.

e Manufactured and/or roll-your-own cigarettes.

Among all current smokers at baseline, 28.3% had quit smoking at follow-up (Table 5). The percentage of recent quitters was higher among participants who initially reported smoking for reasons other than to stress reduction or relaxation (78.5% vs 21.5%, p=0.006); among those who were non-daily smokers in comparison to daily smokers (45.3% vs 17.6%, p<0.001); among those who had low cigarette consumption (<10 CPD) in comparison to those who consumed ≥10 CPD (41.4% vs 18.2% and 19.4%, p<0.001); among those who had low nicotine dependence in comparison those who had medium and high dependence (32.0% vs 19.5%, p=0.036); and among those who had no intention to cut back consumption in comparison to those who intended to reduce consumption (34.7% vs 22.5%, p=0.026).

**Table 5:** Predictors of smoking cessation in a cohort of Catalan nursing students from baseline (2015-2016) to follow-up (2018-2019) according to baseline sociodemographic characteristics and tobacco use patterns (N=276)

	*Recent quitters ^a^*				
	*n*	*%*	*p*	*APR ^b^*	*95% CI*
**Overall**	78	28.3			
**Sex**			0.212		
Male	13	37.1		1.05	0.92–1.21
Female	65	27.0		1.00	
**Age** (years)			0.191	1.00^c^	0.99–1.01
≤19	30	34.1			
20–24	32	23.4			
≥25	15	31.3			
**Year in nursing school**			0.906		
First	28	28.3			
Second	21	31.3			
Third	15	25.4			
Fourth	13	27.7			
**Age at smoking initiation** (years)			0.614		
<17	49	26.8			
≥17	27	29.7			
**Reasons why they started smoking^d^**					
Having a peer/family smoker	48	26.7	0.421		
Other	53	27.5	0.653		
**Reasons why they currently smoke^d^**					
To reduce stress/relax	32	21.5	**0.006**	0.93	0.86–1.02
For pleasure	51	26.6	0.314		
Other	34	25.4	0.301		
**Smoking status**			**<0.001**		
Non-daily smoker	48	45.3		**1.19**	**1.08–1.31**
Daily smoker	30	17.6		1.00	
**Type of tobacco use**			0.222		
Cigarette-only use^e^	59	30.4			
Poly-tobacco use	19	23.2			
**Cigarettes per day**					
<10	48	41.4			
10–19	16	18.2			
≥20	14	19.4			
**Heaviness of smoking index**			**0.036**		
Low (0–2)	62	32.0		1.00	0.91–1.10
Medium and high (3–6)	16	19.5		1.00	
**Quit attempts in the last year**			0.902		
Yes	20	28.6			
No	57	27.8			
**Number of quit attempts in the last year**			0.279		
1	10	33.3			
≥2	7	21.2			
**Are you seriously thinking about quitting now?**					
Yes	11	29.7			
No	64	27.9			
**Are you thinking about cutting back consumption?**					
Yes	34	22.5		0.95	0.88–1.04
No	41	34.7		1.00	

a Compared with ‘continued as smokers’ (n=198). APR: adjusted prevalence ratio.

b PR adjusted for sex, age group, current smoking to reduce stress/relax, smoking status, heaviness of smoking index and thinking about cutting back consumption.

c Continuous variable.

d Multiple responses were accepted.

e Manufactured and/or roll-your-own cigarettes.

### Predictors of tobacco use transition

We found that being a non-daily smoker and having lower nicotine dependence were inversely associated with reducing cigarette consumption (by ≥5 CPD) compared with being a daily smoker (APR=0.33; 95% CI: 0.196–0.55) and having medium and high dependence (APR=0.78; 95% CI: 0.64–0.96) (Table 4). Otherwise, non-daily smoking was the only predictor of quitting smoking (APR=1.19; 95% CI: 1.08–1.31) (Table 5). No effect modification of these predictors was observed in sex- and age-specific analyses.

## DISCUSSION

This study among smoker nursing students provides a longitudinal overview of changes in smokers’ tobacco use patterns over three years and identifies predictors of transitions in tobacco use patterns. The study found that 12.1% of daily smokers at baseline transitioned to non-daily use at follow-up, and more than one-third of non-daily smokers shifted to being daily smokers. Of all the cigarette-only users, 14.2% transitioned to poly-tobacco use, and of all the poly-tobacco users, almost half transitioned to cigarette-only use. Furthermore, among all current smokers (including both daily and non-daily smokers), two-thirds reduced their cigarette consumption by at least 5 CPD, and almost one-third had quit smoking. Finally, whereas being a non-daily smoker and having lower nicotine dependence were inversely associated with reducing cigarette consumption, being a non-daily smoker was directly associated with being a quitter at follow-up.

This range of tobacco use transitions is consistent with existing evidence among college students. This suggests that nursing students, similarly to students in other disciplines, also experience several changes in tobacco use patterns during their training^5,19,20^. Furthermore, a notable proportion of this cohort of nursing students were poly-tobacco and other tobacco product users, with the former being more prevalent among those who were still enrolled in nursing school than among those who had graduated. This finding is consistent with the results of Butler et al.^21^, who found higher odds of poly-tobacco use among lower level undergraduates. Additionally, other research points to young college students being more prone to using alternative tobacco products than older students^22^.

The diverse tobacco use transitions experienced during their college years and the greater prevalence of poly-tobacco use and alternative tobacco product use among university students can be explained by multiple psychosocial factors. First, college students generally seek to experience new sensations, their peers influence them, and they are vulnerable to situations that cause anxiety, which may lead them to consume several emerging tobacco products^23^. Secondly, college students are more exposed to tobacco industry messages than other individuals, which increases their probability of using tobacco products^24^. Finally, social smoking is highly prevalent among college students, which may increase the use of alternative tobacco product use and decrease their perceived addiction^25^. Poly-tobacco and other tobacco product users are as likely as cigarette-only users to intend to quit smoking, which highlights the need to implement tobacco prevention and cessation strategies during college years, especially during the first years, before the consolidation of smoking behaviors^21,22^.

Regarding predictors of tobacco use transition, being a non-daily smoker and having lower nicotine dependence were determinant factors for reducing cigarette consumption and smoking cessation in this cohort of smoker nursing students. Non-daily smokers had a lower probability of reducing their cigarette consumption but a higher probability of quitting smoking. Likewise, the percentage of non-daily users who transitioned to being daily smokers (36.2%) was tripled that of daily smokers who switched to being non-daily smokers (12.1%). Based on these findings, we consider that non-daily smokers in this cohort showed less smoking pattern stability than daily smokers, which is consistent with the results of previous longitudinal studies carried out among college students and other populations^20,26,27^. This lower stability of smoking patterns may be a consequence of the heterogeneity among non-daily smokers who present different behavioral and psychosocial smoking characteristics (frequency and amount of use, social smoking, perceived dependence, etc.)^28^. Although most non-daily smokers have low nicotine dependence, which is a strong predictor of smoking cessation, their lower perceived addiction and other psychosocial factors may inhibit them from reducing cigarette consumption and probably lead to an increase in consumption until they become daily smokers^29,30^.

Although more longitudinal studies are required, these findings highlight the need for a better understanding of potential predictors that may disrupt the pattern of escalating smoking and addiction during the college years, as both non-daily and daily smokers are in a determinant stage to consolidate their tobacco use behaviors. Additionally, current tobacco use behaviors among college students indicate the need to implement tobacco control strategies in universities early and urgently. The implementation of tobacco-free campuses has proven to be effective in the reduction of the overall prevalence of smoking and secondhand exposure among college students. However, their effectiveness may vary by tobacco product^31^. Therefore, a comprehensive enforcement strategy including tobacco-free campus policies, tobacco use prevention and tailored cessation programs, and restrictions on the marketing, advertising, and promotion of tobacco products could be effective in reducing tobacco use among nursing students^3,32^.

### Strengths and limitations

While this study had a large sample at baseline, three-quarters of the participants were lost to follow-up. Those who were male, aged ≥25 years, and current smokers were less likely to participate in the follow-up. In addition, in the definition of daily and non-daily smokers, we only included users of MF and RYO cigarettes, and this may have resulted in low sample sizes in the tobacco use transition groups due to the reduced number of smokers. The small sample size has limited us from analyzing the predictors of all the transitions. While the cohort was restricted to nursing schools in Catalonia, the participants’ characteristics do not appear to differ from those of nursing students from other regions of Spain and Europe^33^.

As far as we know, this is the first longitudinal study in Europe to investigate the predictors of tobacco use transitions in nursing students. The study distinguished between levels of smoking intensity, analyzing non-daily and daily smokers separately. In addition, the survey also explored the use of conventional tobacco products, such as MF and RYO cigarettes and cigars/cigarillos/little cigars, the novel ones, such as e-cigarettes and waterpipes, and cannabis. Finally, although we included several individual and contextual sociodemographic characteristics and variables related to tobacco use patterns as potential predictors of changes in smoking habits, residual confounding.

## CONCLUSIONS

Nursing students who smoked, especially those who were non-daily smokers and poly-tobacco users at baseline, underwent several transitions in their tobacco product use during the follow-up period, either by increasing their consumption, reducing it, or quitting smoking. Being a non-daily smoker and having lower nicotine dependence were inversely associated with reducing cigarette consumption by ≥5 CPD, and only being a non-daily smoker predicted tobacco cessation at follow-up. These findings suggest that tobacco use behavior in this cohort is unstable and emphasize the urgent need for the implementation of a comprehensive strategy to reduce both conventional and novel tobacco product use on university campuses.

## Supplementary Material



## Data Availability

The data supporting this research are available from the authors on reasonable request.
