# Supplementary material for: Determinants of tobacco use transitions in smoker nursing students in Catalonia: A prospective longitudinal study
Source: Tob Induc Dis. 2024 Jul 8;22:10.18332/tid/189484. doi: 10.18332/tid/189484 (PMC11229088; doi:10.18332/tid/189484)
Supplement: Supplementary file 1 [file TID-22-126-s1.pdf]

Table S1: Tobacco use patterns at follow-up (2018−2019) among smokers in a cohort of Catalan nursing students (N=276)

**Characteristics**	**Follow-up smoking status**						***p*-value**[a]
	**Daily smoker**			**Non-daily smoker**			
	n	**%**	**95 CI%**	***n***	**%**	**95 CI%**	
Overall	144	72.7	66.2–78.6	54	27.3	21.4–33.8	
Age at smoking initiation							0.121
<17 years	102	70.8	63.1–77.8	32	59.3	46.0–71.6	
≥17 years	42	29.2	22.2–36.9	22	40.7	28.4–54.0	
Type of tobacco use							0.606
Cigarette-only use[b]	109	76.2	68.8–82.6	36	67.9	54.7–79.3	
Polytobacco use	34	23.8	17.4–31.2	17	32.1	20.7–45.3	
Other product used (in case of polytobacco use)							
Cigars, cigarrillos, little cigars	1	0.7	0.1–3.6	1	1.9	0.2–8.5	1.000
Electronic cigarettes	2	1.4	0.3–4.4	2	3.8	0.8–11.6	0.576
Water pipes	14	9.8	5.7–15.5	13	24.5	14.5–37.2	**0.008**
HTPs[c]	3	2.1	0.6–5.5	1	1.9	0.2–8.5	1.000
Cannabis	20	14.0	9.0–20.4	7	13.2	6.1–24.2	0.888
Cigarettes per day							**< 0.01**
<10	80	55.6	47.4–63.5	53	100	-	
10–19	46	31.9	24.7–39.9	0	0	-	
≥20	18	12.5	7.9–18.6	0	0	-	
Heaviness of smoking index							0.383
Low (0–2)	101	76.5	68.8–83.1	42	100	-	
Medium and high (3–6)	31	23.5	16.9–31.2	0	0	0	
Quit attempts in the last year							0.176
Yes	33	25.0	18.2–32.9	15	35.7	22.6–50.8	
No	99	75.0	67.1–81.8	27	64.3	49.2–77.4	
Number of quit attempts							1.000
1	11	33.3	19.2–50.3	5	33.3	14.0–58.4	
≥2	22	66.7	49.7–80.8	10	66.7	41.6–86.0	
Are you seriously thinking about quitting now?							0.229
Yes	118	89.4	83.3–93.8	33	78.6	64.5–88.8	
No	14	10.6	6.2–16.7	9	21.4	11.2–35.5	
Are you thinking about cutting back consumption?							**< 0.01**
Yes	99	75.0	67.1–81.8	20	47.6	33.1–62.5	
No	33	25.0	18.2–32.9	22	52.4	37.5–66.9	

[a]*Chi-square test (daily smoker vs. non-daily smoker)*

[b]*Manufactured and/or roll-your-own cigarettes*

[c]*Heated Tobacco Products*

Table S2: Follow-up (2018 −2019) sociodemographic characteristics and tobacco use patterns among smokers in a cohort of Catalan nursing students according to type of tobacco use (N=276)

Characteristics	Type of tobacco use at follow-up						
	Cigarette-only use[a]			Polytobacco use			*p*-value[b]
	n	**%**	**95 CI%**	***n***	**%**	**95 CI%**	
Overall	145	74.0	67.5–79.7	51	26.0	20..3–32.5	
Sex							0.511
Male	15	10.3	6.2–16.1	7	13.7	6.4–25.1	
Female	130	89.7	83.9–93.8	44	86.3	74.9–93.6	
Age group							**< 0.05**
<22 years	35	24.5	18.0–32.0	20	39.2	26.7–52.9	
23–24 years	39	27.3	20.5–35.0	22	43.1	30.2–56.8	
≥25 years	69	48.3	40.2–56.4	9	17.6	9.1–29.7	
Finished nursing degree							**< 0.001**
Yes	96	66.2	58.2–73.5	16	31.4	19.9–44.9	
No	49	33.8	26.5–41.8	35	68.6	55.1–80.1	
Occupation							**< 0.001**
Nursing students	40	27.6	20.8–35.3	34	66.7	53.1–78.4	
Nurses	96	66.2	58.2–73.5	16	31.3	19.9–44.9	
Other situations	9	6.2	3.1–11.0	1	2.0	0.8–8.8	
Year of degree (nursing students)							0.119
Second or third	12	30.0	17.6–45.2	5	14.7	5.8–29.3	
Fourth	28	70.0	54.8–82.4	29	85.3	70.7–94.2	
Work area (nurses)							0.471
Hospital	70	81.4	72.2–88.5	10	71.4	45.5–89.5	
Other	16	18.6	11.5–27.8	4	28.6	10.5–54.5	
Type of institution they work in (nurses)							
Public	43	50.0	39.6–60.4	8	57.1	31.9–79.7	0.775
Private or Private with public [illegible]	43	50.0	39.6–60.4	6	42.9	20.3–38.1	
Living status							
With family	86	64.2	55.8–71.9	27	56.3	42.2–69.6	0.331
Independent	48	35.8	28.1–44.2	21	43.7	30.4–57.8	
Household monthly income							0.527
≤€1500	40	27.6	20.8–35.3	19	37.3	25.0–50.9	
€1501–3000	37	25.5	19.0–33.0	12	23.5	13.6–36.4	
>€3000	31	21.4	15.3–28.6	11	21.6	12.0–34.2	
Do not know/Did not answer	37	25.5	19.0–33.0	9	17.6	9.1–29.7	
Marital status							0.052
Single	90	67.7	59.4–75.2	40	83.3	71.0–91.8	
Other	43	32.3	24.8–40.6	8	16.7	8.2–29.0	
Cigarettes per day							0.668
<10	99	68.8	60.9–75.9	33	64.7	51.1–76.7	
10–19	31	21.5	15.4–28.8	14	27.5	16.7–40.7	
≥20	14	9.7	5.7–15.4	4	7.8	2.7–17.6	
Heaviness of smoking index							0.807
Low (0–2)	103	81.7	74.3–87.7	40	83.3	71.0–91.8	
Medium and high (3–6)	23	18.3		8	16.7	8.2–29.0	
Quit attempts in the last year							0.154
Yes	31	24.6	17.7–32.6	17	41.2	23.1–49.5	
No	95	75.4	67.4–82.3	31	64.6	50.5–76.9	
Number of quit attempts							0.393
1	9	29.0	15.4–46.3	7	41.2	20.7–64.4	
≥2	22	71.0	53.7–84.6	10	58.8	35.6–79.3	
Are you seriously thinking about quitting now?							0.407
Yes	111	88.1	81.6–92.9	40	83.3	71.0–91.8	
No	15	11.9	7.1–18.4	8	16.7	8.2–29.0	0.505
Are you thinking about cutting back consumption?							
Yes	88	69.8	61.4–77.3	31	64.6	50.5–76.9	
No	38	30.2	22.7–38.6	17	35.4	23.1–49.5	

[a]*Manufactured and/or roll-your-own cigarettes*

[b]*Chi-square test (cigarette-only use vs. polytobacco use)*

©2024 Laroussy K. et al.
